# Fatty Acid Methyl Esters From the Coral-Associated Bacterium *Pseudomonas aeruginosa* Inhibit Virulence and Biofilm Phenotypes in Multidrug Resistant *Staphylococcus aureus*: An *in vitro* Approach

**DOI:** 10.3389/fmicb.2021.631853

**Published:** 2021-03-23

**Authors:** Karuppiah Vijay, George Seghal Kiran, S. Divya, Kavitha Thangavel, Sathiamoorthi Thangavelu, Ranjithkumar Dhandapani, Joseph Selvin

**Affiliations:** ^1^Department of Microbiology, School of Life Sciences, Pondicherry University, Puducherry, India; ^2^Department of Microbiology, School of Biological Sciences, Alagappa University, Karaikudi, India; ^3^Department of Food Science and Technology, School of Life Sciences, Pondicherry University, Puducherry, India

**Keywords:** coral bacteria, *Staphylococcus aureus*, antibiofilm, antivirulence, antibaceterial, *Favites* sp.

## Abstract

In an attempt to study the antibacterial, antivirulence and antibiofilm potentials of bacteria residing the tissue and surface mucus layers of the pristine corals, we screened a total of 43 distinct bacterial morphotypes from the coral *Favites* sp. Among the isolates, *Pseudomonas aeruginosa* strain CBMGL12 with showed antibacterial, antivirulence and antibiofilm activity against multidrug resistant pathogenic strains of *Staphylococcus aureus* (reference strain: MTCC96; community-acquired methicillin resistant strain: CA-MRSA). Extracellular products (ECP) from the coral-associated bacterium *P. aeruginosa* were solvent extracted, fractionated by chromatographic techniques such as silica column and HPLC-UV with concomitant bioassays guiding the fractionation of metabolites. Identification of bioactive chemical moieties was performed by FT-IR analysis, GC-MS/MS equipped with NIST library, ^1^H and ^13^C NMR spectral studies. We report the differential production of extracellular and cell-associated virulence and biofilm phenotypes in multi-drug resistant strains of *S. aureus*, post-treatment with the ECP containing aromatic fatty acid methyl esters (FAME) such as methyl benzoate and methyl phenyl acetate produced by a coral-associated bacterium. In conclusion, this study has identified antibacterial, antibiofilm and antivirulent FAME from the coral-associated *P. aeruginosa* for its ability to attenuate virulence and biofilms phenotypes in multi-drug resistant pathogenic strains of *S. aureus.*

## Introduction

*Staphylococcus aureus* is remarkably a versatile microorganism that adapts tremendous interactions to its niche. It does exist on inanimate sites and animate hosts. For an instance, harmless as a commensal flora of the skin or mucous membranes; as an opportunistic pathogen for a variety of body tissues attributing several diseased conditions ranging from minor skin infections to toxicosis and as a potential drug-resistant pathogen causing systemic, life-threatening illnesses. *S. aureus* is highly regarded to be a human pathogen causing a broad range of illnesses to humanity. Infection can be either nosocomial or community-acquired in humans. Illness includes toxin-mediated diseases like Toxic Shock Syndrome (TSS), septic shock and other focal infections include infective endocarditis, bacterial arthritis, and pneumonia (Ferry et al., 2005). This kind of versatility may be due to a range of adaptive or accessory gene systems (Cramton et al., 1999; Stewart and Costerton, 2001; Baba et al., 2002; Fux et al., 2005). The accessory genetic system conferring pathogenesis in *S. aureus* is regulated by a complex network of two-component system named *agr* system, a quorum-sensing (QS) regulon, which is distributed as four variants forming four different QS groups of *S. aureus* strains. Accessory gene regulator (*agr*) locus encodes the components involved in the QS system of *S. aureus* (Novick, 2003). In *S. aureus*, *agr* system regulates the expression of virulence genes in response to an autoinduction by autoinducing peptides (AIPs) and result in cell dispersal from established Staphylococcal biofilms with the help of extracellular proteases (Mayville et al., 1999; Lyon et al., 2002; Dong et al., 2007; Boles and Horswill, 2008, 2011). It implies one has to keep track of the expression of virulence phenotypes and biofilm-forming potential in several multi-drug resistant (MDR) strains of *S. aureus* in order to explore a novel antivirulence strategy.

Coral reefs provide a suitable ecosystem for microbial interactions through signaling (Pearse and Muscatine, 1971; Rowan and Knowlton, 1995; Stanley and Swart, 1995; Gowrishankar et al., 2012; Krediet et al., 2013). Therefore, the need for finding novel bioactive molecules that can degrade/interfere with QS signals of virulent *S. aureus* may be satisfied by coral-associated bacterial populations that habitually compete with other natural biofilm formers on corals. The coral-Associated bacterium *Bacillus firmus* as the most promising source of antibiofilm agents targeting the recalcitrant biofilms of multidrug resistant-MRSA and MSSA (Gowrishankar et al., 2012), but the chemical identification of the bioactive metabolite was lacking. Gordon et al. (2013) reviewed several natural molecules and their synthetic analogs, which could attenuate the virulence gene expression of *S. aureus* in four different modes such as competitive inhibitors of AgrC–AIP interaction; inhibitors of AgrA–DNA interactions; small molecule inhibitors affecting RNAIII expression; and inhibitors of transcriptional regulators. Mansson et al. (2011) reported the production of cyclodepsipeptides such as solonamides A and B from the marine mussel surface bacterium *Photobacterium halotolerans* and their ability to interfere with *agr* QS system of *S. aureus* and the peptides were able to inhibit the expression of virulence genes in a gene reporter-agar diffusion assay. However, the drug resistance and biofilm potentials of the *S. aureus* strains used were not explored in their study. Besides, the preparations of cyclodepsipeptides for environmental and biomedical applications to control biofilms formed by MDR pathogenic *S. aureus* is quit critical unlike the fatty acid methyl esters from bacteria of pristine ecological niche like corals. Because the biofilm and adherence genes play pivotal role in enhancing the virulence of *S. aureus* (Nourbakhsh and Namvar, 2016), it is essential to study antibiofilm and antivirulence ability simultaneously in order to explore active molecules with multipotency.

Though the recent decade has collections of scientific studies pertaining to the exploration of novel bioactive molecules from marine bacterial resources, their precise targeting on multi-drug resistant and biofilm forming *S. aureus* accomplished with the determination of involved chemical groups are scanty. Herein, we studied the antibacterial, antivirulence and antibiofilm potentials of FAME secreted by the coral-associated bacterium *Pseudomonas aeruginosa* targeting MDR *S. aureus* and chemically characterized the extracellular bioactive components of interests by spectral and GC-MS/MS analysis.

## Materials and Methods

### Properties of Pathogenic Bacterial Strains Used

*S. aureus* strain was collected from a clinical lab in Puducherry, India, and was used as a test pathogen in this study. *S. aureus* MTCC96 was used as a pathogenic reference strain. The test strain was screened for multidrug resistance potential in an antibiotic susceptibility testing by Kirby-Bauer disk diffusion assay (Bauer et al., 1966). Concentration of each antibiotics used was as per the guidelines from CLSI (Wikler, 2006). Phenotypic expression of extracellular virulence factors such as hemolysin α/β and proteases were studied using enriched plate assays for both strains (Kloos and Schleifer, 1975; McNamara et al., 2000; Miedzobrodzki et al., 2002). Potential to form biofilms *in vitro* was evaluated by modified tube assays and modified MTP assays (Stepanovic et al., 2000).

### Isolation of Coral-Associated Bacteria (CB)

Sterile needleless syringes, swabs, and chisels were used to collect the surface mucus layer and tissue specimen of the coral *Favites* sp. at Palk bay, Mandapam North Sea outside Gulf of Mannar Marine National Park (9.3°S, 79.1°E), Ramanathapuram, Tamil Nadu, India, using the method adopted by Thinesh et al. (2020). Collected samples were immediately brought to the laboratory in sterile aged seawater (SAS) for further processing (Kiran et al., 2010). Tissue was homogenized with SAS using precooled sterile mortar and pestle. Both surface mucus layer and ground tissue samples in SAS were serially diluted to 10^–7^ folds and spread onto nutrient agar plates supplemented with 2% NaCl at pH adjusted to 7.8, Luria Bertani agar at pH adjusted to 7.8, Zobell Marine agar, and incubated at 30°C for 24 h to isolate coral-associated bacterial colonies (Gowrishankar et al., 2012).

### Solvent Extraction of Extracellular Metabolites

For extraction of extracellular metabolites, coral-associated bacterial colonies were cultured in nutrient broth supplemented with 2% NaCl at pH 7.8 and incubated under shaking (150 rpm) conditions for 24–72 h at 30°C. Grown cultures were pellet down at 12,000 rpm for 10 min at 4°C to collect cell-free supernatants. All the supernatants were filtered through 0.2 μm filter, acidified using concentrated HCl to reduce the pH to 2.0 facilitating the protonation of water molecules releasing bioactive compounds. The acidified supernatants were twice extracted with an equal volume of EtOAc (Kiran et al., 2010). The resultant EtOAc extracts were concentrated to dryness using a rotary vacuum evaporator (R-300, BUCHI Corporation, Switzerland) and the concentrate was re-dissolved separately in different solvents such as water, dimethyl sulphoxide, methanol, ethyl acetate, and chloroform to assess the polarity. The extract was soluble in methanol, chloroform, and ethyl acetate, indicating that the active molecules were slightly non-polar to mid-polar in charge. However, for toxicity reasons, the ethyl acetate extracts were screened for further studies.

### Screening for Antibacterial Potentials

For evaluation of antibacterial activity, agar well diffusion assay was employed using Muller-Hinton Agar (MHA). Overnight cultures of clinical isolates were subcultured in Tryptic Soy Broth (TSB) until turbidity of 0.5 McFarland (1 *×* 10^8^ CFU/mL) was observed. Pathogens were uniformly spread across the surface of the agar plate using sterile cotton swabs. In the swabbed plates, agar wells were punched with a diameter of 6 mm; loaded with coral-associated bacterial extracts, and EtOAc (negative control) at the concentration 100 *μ*l/well, and the plates were incubated at 37°C to observe the zone of lysis after 24 h (Gowrishankar et al., 2012). Only the extracts which exhibited a prominent zone of growth inhibition against the test and MTCC96 *S. aureus* were used for further study.

### Antibiofilm Potential in 96-Well Microtitre Plate Assay

The effect of coral-associated bacterial extracts on biofilms of *S. aureus* strains was evaluated in 96-well flat bottomed polystyrene plates. Wells added with 10 μL of Staphylococcal cell suspensions prepared in 1× phosphate-buffered saline (PBS) and with each of the CB extracts (in EtOAc) in volume ranging from 10–50 μL were treated as test wells. Wells filled with 10 μL each of Staphylococcal cell suspensions and EtOAc in volume ranging from 10–50 μL was treated as the control. Wells added with 10 μL of 1× PBS, and each of the CB extracts in volume ranging from 10–50 μL served as blanks for the respective test and positive control wells. All the test, control, and blank wells were made up to a final volume of 200 μL using TSB. The experimental set up was prepared in triplicates and incubated for 24 h at 37°C. The biofilms formed were stained with 0.1% crystal violet in water (w/v) for 5 min (Kiran et al., 2010; Gowrishankar et al., 2012). The unbound stain was discarded, and the biofilms were washed twice with sterile deionized water followed by air drying. The optical density was measured at 595 nm (Kiran et al., 2010), and the level of biofilm inhibition was marked in terms of percentage using the following formula:

Percentage of

Inhibition (%) = [(Control OD595 nm − Test OD595 nm)/Control OD595 nm] × 100

The biofilm inhibitory concentration (BIC) was measured as the lowest concentration which produced a noticeable disruption of biofilms under stereo zoom microscopy and a significant reduction in the OD value at 595 nm when compared with its respective control well. Biofilm inhibitory potential was confirmed in fluorescence microscopy (blue filter for excitation wavelength with emission in green fluorescence) after staining with 0.1% acridine orange.

### Effect of the CB Extract on Cell Density of *S. aureus* Strains

The effect of coral-associated bacterial extracts on bacterial cell density was assessed in 96-well polystyrene plates. Wells containing TSB were inoculated with test and reference pathogens. All the wells were loaded with coral-associated bacterial extracts at MIC and sub-MIC, and incubated at 37°C for 24 h (Andrews, 2001). Wells without extracts but inoculated with pathogens served as a control, and the medium with extract served as the blank. After incubation, the contents were gently tapped, and the cell population was quantified using spectrophotometry at 600 nm (Gowrishankar et al., 2012). A viable plate count was also performed to correlate with the reduction in the optical density values of the pathogenic strains of *S. aureus* as compared to the untreated controls.

### Screening for Anti-virulence Potential

Coral-associated bacterial extracts which exhibited antibacterial and antibiofilm properties were further screened for their ability to inhibit the production of secretory virulence factors such as hemolysin α/β, proteases as well as the cell-associated virulence factors of *S. aureus* strains used in this study.

#### Production of Extracellular Virulence Factors

A 1% inoculum of reference and test pathogens was added to the MHB supplemented with 0.5% polysorbate 80 both in the presence and absence of CB extract at MIC and incubated at 37°C under shaking until the OD at 600 nm reached a value of 2.5. After centrifugation at 10,000 rpm twice, the supernatants containing secretory virulence factors were recovered from the pathogens (incubated with extract at MIC) and used for further assays (Gowrishankar et al., 2012).

#### Total Hemolysin Inhibition Assay

Hemolytic activity of the test and reference strains was initially observed on blood agar plates (Kloos and Schleifer, 1975). A total of 2% sheep erythrocytes prepared in 1× PBS was added to the buffer containing 10 mM Tris, 160 mM NaCl, and 20 mM CaCl_2_ adjusted to pH 7.4 using HCl. A total of 300 *μ*L of extracellular products (ECP) produced from *S. aureus* strains were added to 2700 *μ*L of 2% sheep RBCs and incubated at 37°C for 30 min. After incubation, the mixture was kept on ice for 20 min followed by centrifugation around 12,000 rpm at 4°C. The released hemoglobin in the supernatant was estimated by absorbance at 530 nm (Gowrishankar et al., 2012). The results were represented in terms of the percentage of hemolysin inhibition for the test (pathogens treated with CB extract) compared to control (pathogens untreated with CB extracts).

#### Protease Inhibition Assay

Proteolytic activity due to the production of extracellular proteases from test and reference *S. aureus* strains was confirmed on skim milk agar plates incubated at 37°C for 24 h (McNamara et al., 2000; Miedzobrodzki et al., 2002). In order to evaluate the protease inhibition, wells with a diameter of 6 mm were cut onto the skim milk agar plates and loaded with aliquots of exoproteins from the test (treated with extract) and control (without extract treatment). Post loading, the plates were incubated at 37°C for 24 h.

#### FTIR Analysis to Study Changes in Cell-Associated Biomolecules

To study changes in cell-associated virulence factors, both test and reference *S. aureus* strains were cultured in TSB along with extracts at MIC until the OD values of respective controls (pathogens free from extract treatment) reached 2.5 at 600 nm. After incubation, cells were pelleted down by centrifugation at 10,000 rpm and dried. The potassium bromide (KBr) pellet technique was adopted for FT-IR analysis (Gowrishankar et al., 2012). FT-IR spectrum was recorded in the IR spectral region between 4,000 and 400 cm^–1^ (Thermo Nicolet-6700).

### Phylogenetic Analysis of the Coral-Associated Bioactive Bacterium

For molecular taxonomic identification, the genomic DNA from CBMGL12 was extracted using standard procedures adopted from the molecular cloning laboratory manual by Green and Sambrook (2012). The quantity and quality of extracted DNA was assessed using NanoDrop spectrophotometer (ND 1000). 16S rRNA gene was amplified using the GeneAmp PCR System 9700 (Applied Biosystems). 16S gene amplicons were initially purified by ExoSAP-IT kit (USB). The purified amplicons were sequenced. The gene sequence of the coral-associated bacterium was submitted in GenBank to obtain the accession number. The phylogenetic analysis was performed using Mega software version 6.0 to establish molecular evolutionary relationships (Felsenstein, 1985; Tamura, 1992; Tamura et al., 2013).

### Production and Purification of Bioactive Molecules

The strain CBMGL12 was mass produced in 1,000 mL Erlenmeyer flask containing 600 mL of nutrient broth supplemented with 2% NaCl (at pH 7.8) and incubated at 30°C for two days at 150 rpm. The culture broth was pellet down twice at 6,000 rpm for 15 min at 4°C, and the cell-free supernatant was collected. The supernatant was acidified with concentrated HCl to achieve the final pH 2.0 and extracted twice with an equal volume of EtOAc (Kiran et al., 2010). The crude solvent extract was concentrated using a rotary vacuum evaporator (R-300, BUCHI Corporation, Switzerland).

The concentrated extract was resolved initially on TLC plates using silica gel 60 Gas stationary support (Stahl, 1969) and different combinations of solvents with varying polarity as mobile phase (Landgrebe, 1977; Lade et al., 2014) to study the number of spots having active fractions (Kiran et al., 2010) and also to identify a suitable solvent system for next step purification using column chromatography. After observation of characteristic pattern of spots on TLC, the crude EtOAc extract was purified through column chromatography using chloroform: EtOAc as a solvent system and silica gel (mess size 100–200) as stationary support. The column purified active fraction was further fractionated through the C18/ODS column of preparative HPLC-UV (UFLC-Shimadzu) equipped with a photodiode array detector. The solvent system used in liquid chromatography was chloroform : methanol (HPLC grade) in the following ratios 95: 5, 90: 10, 85:15, and 80:20 with a flow rate of 0.5 ml/min (Mansson et al., 2011). All the purified fractions were subjected to the above discussed antivirulence assays in order to confirm the presence of active molecules.

### Chemical Characterization of HPLC Purified Bioactive Fractions

HPLC purified anti-virulent fraction was analyzed through FT-IR and GC-MS/MS equipped with NIST library to study the associated chemical moieties and to predict the presence of different types of fatty acid esters, respectively. The structural elucidation of the active fraction was carried out through Fourier Transform Nuclear Magnetic Resonance spectroscopy (400 MHz-FT-NMR, Bruker-Advance-II, Germany). ^13^C (100 MHz) and proton (400 MHz) NMR spectra were recorded after overnight scanning runs (TOPSPIN NMR data system).

## Results

### Multidrug Resistance, Virulence and Biofilm Phenotypes in Pathogenic *S. aureus* Strains

**FIGURE 1:**
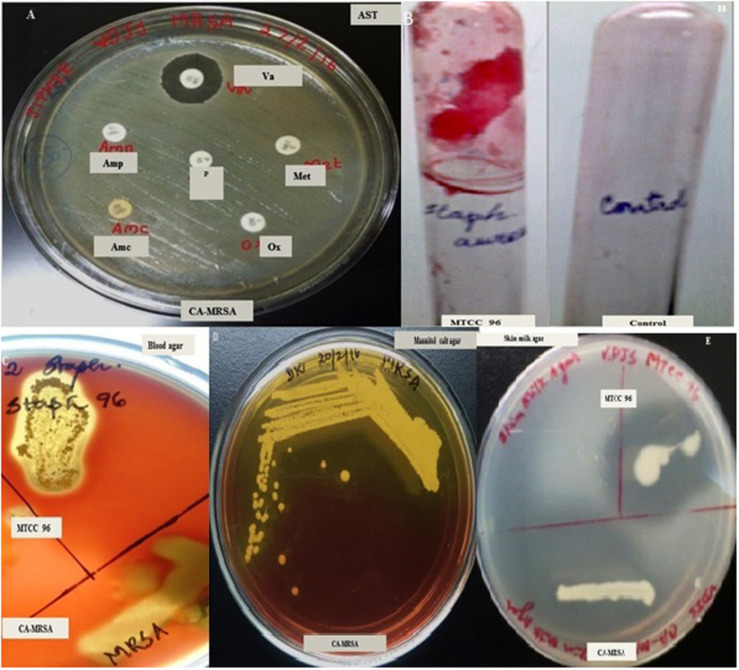
Screening for multidrug resistance in *Staphylococcus aureus* strains [CA-MRSA **(A)**] using Kirby-Bauer Disc diffusion assay. **(B)** Recalcitrant biofilm formed by CA-MRSA in modified tube assay. **(C)** Hemolytic activity of MTCC96 and CA-MRSA on Blood agar, **(D)** CA-MRSA on Mannitol Salt Agar, **(E)** Protease activity of MTCC96 and CA-MRSA on Skim milk agar. AST, Antibiotic Sensitivity Testing; Va, Vancomycin (30 μg/disc); Met, Methicillin (10 μg/disc); P, Penicillin (10 units/disc); Amp, Ampicillin (10 μg/disc); Amc, Amoxicillin with clavulanic acid (20/10 μg per disc); Ox, Oxacillin (1 μg/disc).

The pathogenic *S. aureus* test strain responsible for community-acquired infections was resistant to Penicillin (10 units), Ampicillin (10 μg), Amoxicillin/clavulanic acid (20/10 μg), Oxacillin (1 μg), and Methicillin (10 μg) but found sensitive to Vancomycin (30 μg) as identified by Kirby-Bauer antibiotic disc diffusion assays (Figure 1A). The test strain was designated as CA-MRSA (Figure 1D). CA-MRSA exhibited extracellular virulence phenotypes such as hemolysin β and proteases, as confirmed by enriched agar plate assays. The potential to develop biofilm *in vitro* was absent in CA-MRSA as observed by modified tube and microtitre plate assays. On the other hand, the reference strain MTCC96 was able to develop a recalcitrant biofilm *in vitro* (Figure 1B) as observed in modified tube/microtitre plate assays and also produced the secretory virulence factors such as hemolysin α (Figure 1C) and proteases (Figure 1E) as identified in enriched agar plate assays. Hence, the two *S. aureus* strains were tested for further antibacterial, antivirulence and antibiofilm studies.

### Screening for Antibacterial, Antibiofilm Potentials

**FIGURE 2:**
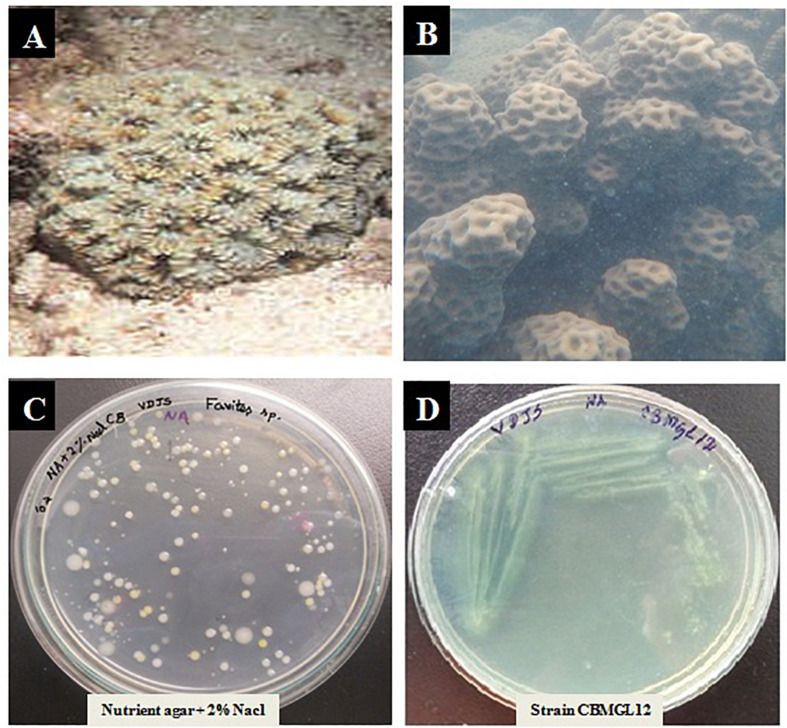
Isolation of Bacteria from the coral *Favites sp.* **(A)** Underwater view of the coral *Favites* sp. outside the Gulf of Mannar Marine Biosphere Reserve. **(B)** *Favites* sp. after sampling the tissue and mucous using needleless syringe and swabs. **(C)** Isolated colonies of coral-associated bacteria (CB) on nutrient agar (NA) plate with 2% NaCl. **(D)** Pure culture of bioactive isolate CBMGL12 on NA plate.

**FIGURE 3:**
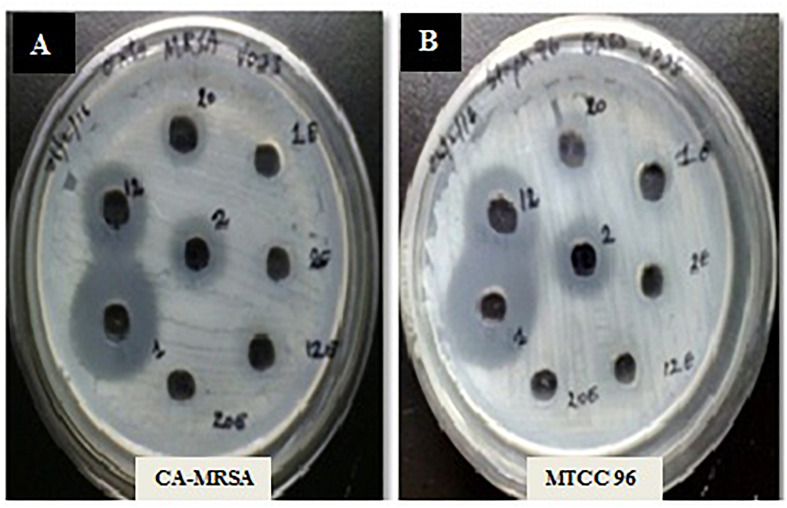
Antibacterial activity of the CFS against *S. aureus* strains. MHA plates representing the antibacterial activity of CFS from 48 h grown cultures of CB-1, 2, and 12 against CA-MRSA **(A)** and MTCC96 **(B)**.

**FIGURE 4:**
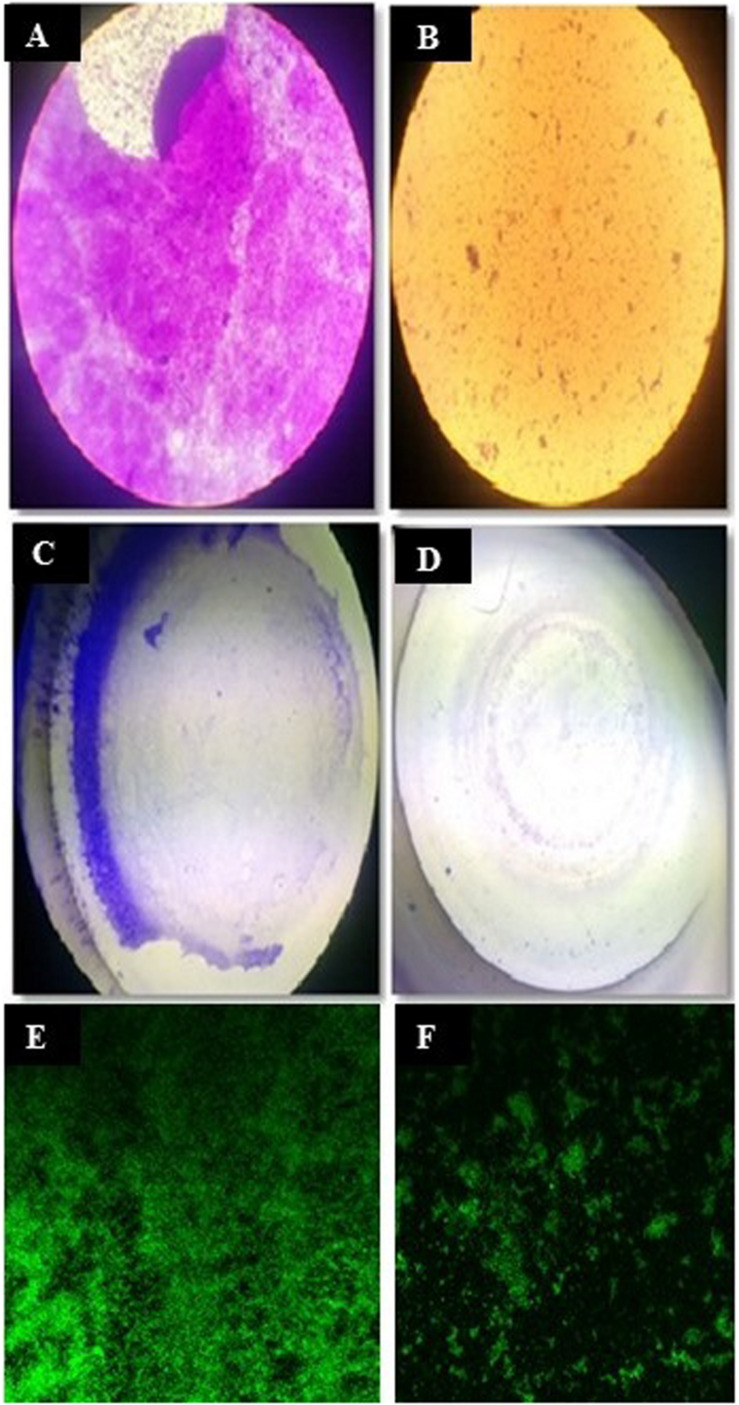
Antibiofilm assay. Light microscopic view of *S. aureus* MTCC96 biofilm **(A)** and its disruption by CBE12 extract at MIC **(B)**. Stereozoom microscopic view of MTCC96 biofilm **(C)** and its disruption by CBE12 extract at MIC **(D)** in MT plate wells. Fluorescence microscopic view of acridine orange stained, untreated **(E)** and CBE12 extract treated **(F)** biofilm of *S. aureus* MTCC96.

**FIGURE 5:**
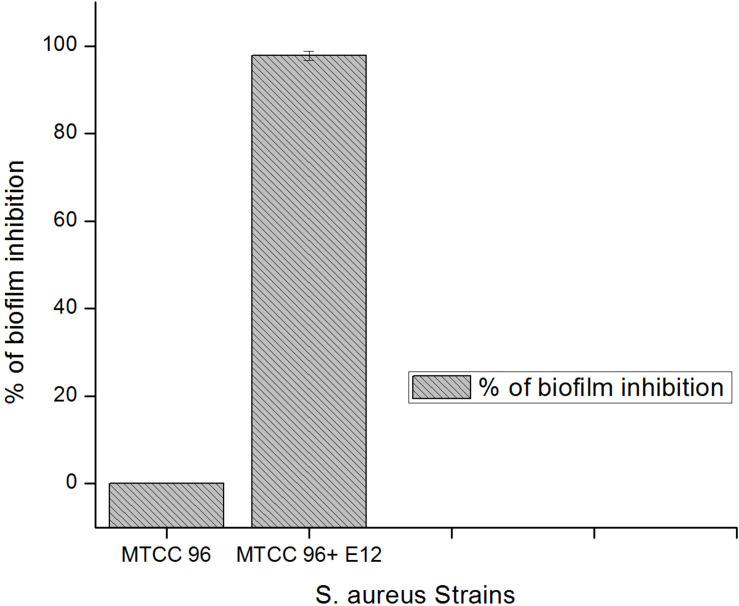
Percentage of biofilm inhibition by CBE12 at BIC (50 μL) in 96-well MT plate as derived from OD values (triplicates) at 595 nm and compared to its untreated control. CBE12 is given as E12 in the figure.

**FIGURE 6:**
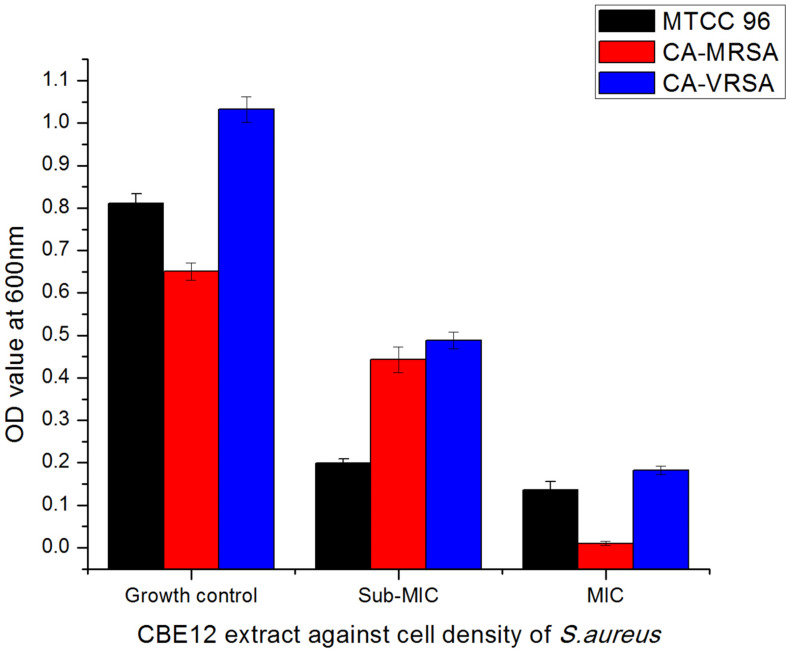
Effect of CBE12 on cell density of *S. aureus* strains at MIC (50 μL) and sub-MIC (25 μL). CA-VRSA strain did not express virulence factors and biofilm properties *in vitro*. Hence, the strain was not taken for the later experimentations.

A total of 43 morphologically distinct bacterial colonies were isolated from the surface mucus layer and ground tissue samples of the coral *Favites* sp. (Figure 2). The coral-associated bacterial isolates were designated from CBMGL1 to CBMGL43. Out of the 43, cell-free supernatants from the three bacterial isolates (CBMGL1, CBMGL2, CBMGL12) exhibited prominent zone of growth inhibition against *S. aureus* strains in an agar well diffusion assay when loaded at 100 μL/well (Figure 3). However, when extracted with an equal volume of ethyl acetate, the extract CBE 12 (from CBMGL12) exhibited antibacterial activity against CA-MRSA and MTCC96 (Supplementary Figure 1). The metabolite production was enhanced during nutrient limiting conditions, and a maximum was achieved after 72 h of growth at pH 7.8, 30°C, under constant shaking at 150 rpm. The MIC of CBE12 was found to be 50 and 25 μL against MTCC96 and CA-MRSA, respectively, as revealed in broth tube dilution technique. Besides, the extract exhibited significant inhibition of biofilms formed by MTCC96 (Figures 4, 5) when viewed under stereo zoom and fluorescence microscopy. CBE12 extract was able to reduce the total planktonic bacterial cell density in both strains of *S. aureus* (CA-MRSA and MTCC96) at its MIC and sub-MIC when compared with respective untreated growth controls (Figure 6). Hence, the extract CBE12 was taken to screen for the associated antivirulence potentials.

### Screening for Antivirulence Potentials

Initially, the percentage of RBC lysis was estimated from the released hemoglobin OD at 530 nm in control (ECP) and test (Supplementary Figure 2). From the percentage of RBC lysis, the percentage of hemolysis inhibition was deduced as an indicative of the inhibition of hemolysin production in the pathogens. Percentage of hemolysis inhibition was found to be 70.59 and 78.97% on MTCC96 and CA-MRSA, respectively (Table 1). Protease inhibition was confirmed on the skim milk agar plate as the reduced zone of casein hydrolysis around the wells loaded with ECP from CBE12 treated CA-MRSA and CBE12 treated MTCC96 when compared with respective control wells (Supplementary Figure 3).

**TABLE 1:** Percentage of hemolysis inhibition by the extract CBE12.

CBE12 treated *S. aureus* strains	Control	% of RBC lysis after CBE12 treatment	% of hemolysis inhibition by CBE12
MTCC 96	100	29.41	70.59
CA-MRSA	100	21.03	78.97

**FIGURE 7:**
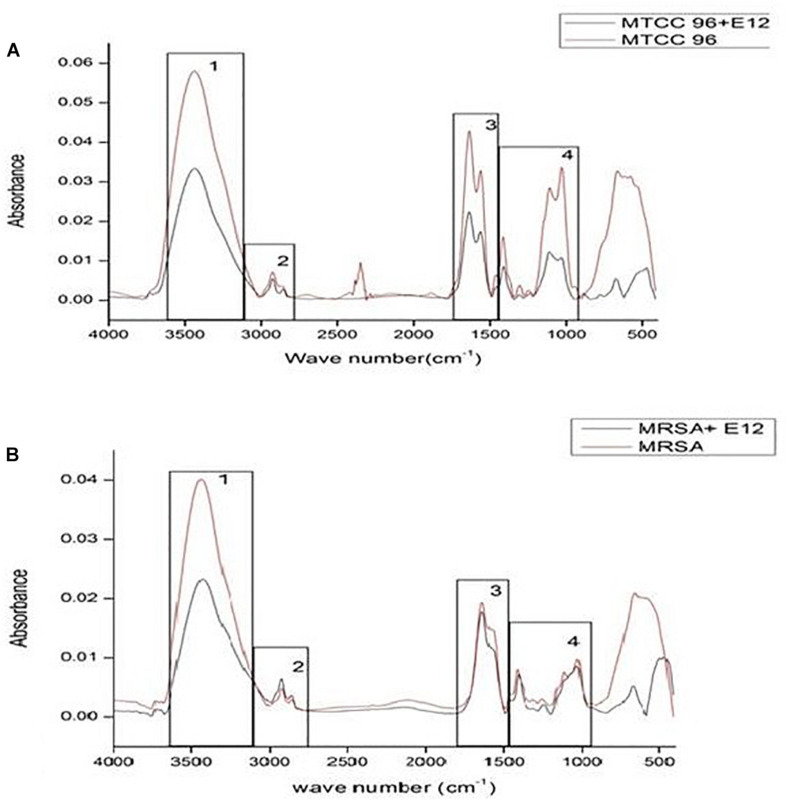
FT-IR spectrum to study the effect of CBE12 on the cell-associated virulence factors of MTCC 96 **(A)** and CA-MRSA **(B)**.

In an attempt to observe the inhibition of cell-associated virulence factors, FT-IR spectral studies were carried out for CBE12 treated and untreated (control) MTCC96 and CA-MRSA cell pellets using KBr pelleting technique. FT-IR spectra were recorded after all the baseline corrections. CBE12 exhibited a marked reduction in the production of cell surface virulence proteins in both MTCC96 (Figure 7A) and CA-MRSA (Figure 7B). Besides, CBE12 showed a reduction in membrane fatty acid moieties of MTCC 96 when compared to the respective control cell pellet (untreated). On contrary, the membrane fatty acid moieties were increased in CA-MRSA.

### Phylogenetic Analysis of Coral-Associated Bioactive Bacterium CBMGL12

**FIGURE 8:**
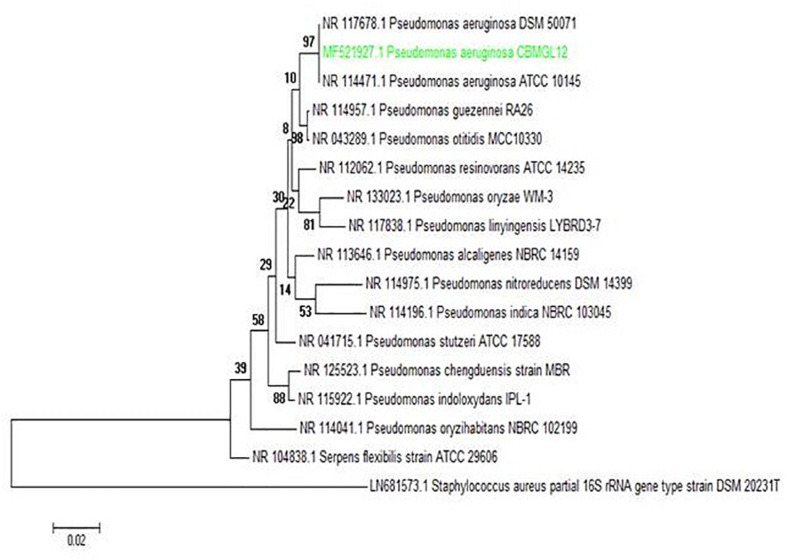
Phylogenetic analysis of the producer bacterium *Pseudomonas aeruginosa* strain CBMGL12. The evolutionary history was inferred by using the Maximum Likelihood method based on the Tamura 3-parameter model. Scale bar represents nucleotide substitutions per site.

Standard biochemical analysis showed the bioactive strain CBMGL12 as *Pseudomonas* sp. (Supplementary Table 1). Molecular taxonomic identification of CBMGL12 revealed the bioactive bacterium as *P. aeruginosa* (GenBank accession number: MF521927) through 16S rRNA gene sequencing. Phylogenetic analysis was conducted using MEGA software version 6.0. The phylogenetic tree was constructed using the UPGMA method. The tree with the highest log likelihood (−4179.1541) was shown in Figure 8.

### Purification and Characterization of Antivirulence Fatty Acid Methyl Esters

**FIGURE 9:**
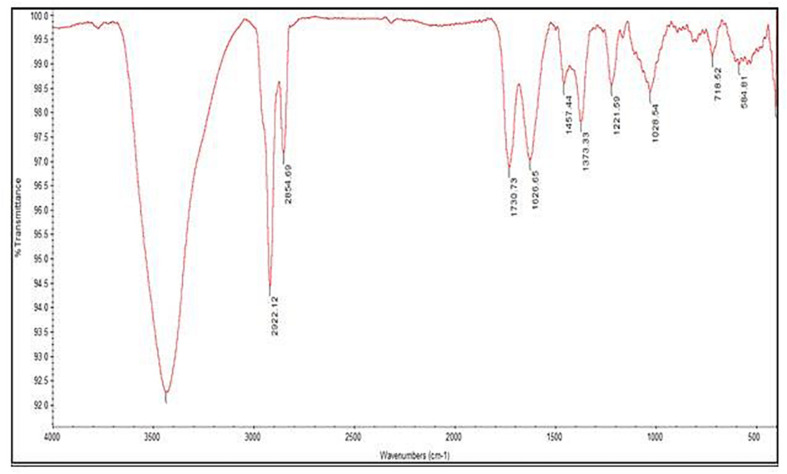
FT-IR spectrum of antivirulence fraction separated by column chromatography.

UV-visible spectral analysis of the active crude extract records the absorbance maxima between 200 to 300 nm indicating the polygenic nature of the CBE12 extract. The crude extract CBE12 was fractionated by column chromatography. The column purified active fraction (CFA2, 10:90 (v/v) EtOAc/CHCl_3_) was further characterized using HPLC-UV, TLC, FT-IR, and GC-MS/MS analyses. In the silica gel TLC, three discrete spots were observed with the R_*f*_ value of biologically active spot as 0.57. Bioassay directed fractionation was performed in TLC and HPLC (Supplementary Figure 4 and Supplementary Tables 2, 3). The FT-IR (Figure 9) and GC-MS/MS (Table 2) analyses revealed fatty acid methyl esters such as methyl benzoate and methyl phenyl acetate may be responsible for antibiofilm and antivirulence activity. The proton NMR data marks the presence of proton types in methyl ester group at 3.686 ppm and the protons of participating carbon atom of benzene rings linked to acetic acid methyl ester at 7.8 ppm (Ortho), 7.6 ppm (Meta), and 7.7 ppm (Para). Proton peak at 8.05 ppm suggests the proton type link to the ortho carbons of benzoic acid methyl ester. ^13^C NMR data indicates the occurrence of carbon types in the benzene ringed structures at 127.2 ppm (Meta), 128.7 ppm (Ortho), 129.5 ppm (Para). Overall, the NMR spectra suggest the presence of compounds having aromatic rings linked to the methyl ester groups in purified bioactive fraction which could possibly be involved in the antibiofilm and antivirulence activities of the coral-associated bacterial extract (Supplementary Figures 5, 6).

**TABLE 2:** Antibiofilm, antivirulence compounds from coral-associated bacterium *Pseudomonas aeruginosa* strain CBMGL12 as predicted by GC-MS/MS analysis assisted with NIST library.

Sl.no.	Peak name	Retention time	% Peak area	Structure
1	Name: benzoic acid, methyl ester Formula: C_8_H_8_O_2_ MW: 136	17.812	1.12	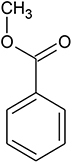
2	Name: benzene acetic acid, methyl ester Formula: C_9_H_10_O_2_ MW: 150	21.720	13.66	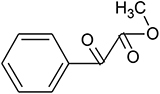

## Discussion

In this study, the extracellularly released bioactive compounds from coral-associated bacterium *P. aeruginosa* were extracted with ethyl acetate after acidifying the cell-free supernatant to pH 2.0 with 1N HCl in order to facilitate the protonation of water molecules which in turn releases the active molecules into the solvent phase. QS is a population dependent mechanism, a decrease in cell density is usually expected at MIC and sub-MIC of the CB extracts. It is evident in our study that the extract CBE12 exerted a steady reduction in viable colony count as well as the cell density of both *S. aureus* strains as indicated by a gradual decrease in growth OD of MTCC96, and CA-MRSA strains treated at sub-MIC and MIC when compared to the corresponding untreated controls.

Biofilm inhibition was observed in MTCC96 when treated with the extract CBE12 at BIC. Though the extract CBE12 inhibited the production of secretory virulence proteins such as hemolysins and proteases and also the cell surface distributed virulence proteins in both *S. aureus* strains, the same extract slightly increased the membrane fatty acid moieties of CA-MRSA strain as observed in FT-IR analysis of extract treated *S. aureus* cell pellets.

When investigating the chemical nature of HPLC purified, active fractions of the secondary metabolites from coral-associated bacterium using FT-IR analysis and GC-MS/MS equipped with NIST library, we observed the likely involvement of aromatic fatty acid methyl esters (FAME) such as methyl benzoate and methyl phenyl acetate, in the inhibition of virulence and biofilm phenotypes in *S. aureus*. NMR data suggest the presence of participating carbon atoms of benzene ringed structures in ^13^C NMR and the proton types of methyl ester and phenyl groups in ^1^H NMR. As reported previously (Gordon et al., 2013), the compounds which have benzene ringed derivatives with carbonyl moieties could act as virulence factor inhibitors and interfere with the QS system in *S. aureus*. Another study in which the phenyl rings and carbonyl groups of cyclodepsipeptides such as solonamides A and B of marine mussel surface bacterium, *Photobacterium* were responsible for the inhibition of virulence gene expression in *S. aureus* through their interaction with AgrC, a receptor histidine kinase (Mansson et al., 2011). Therefore, the two fatty acid methyl esters such as methyl benzoate and methyl phenyl acetate produced by the coral-associated bacterium *P. aeruginosa* strain CBMGL12 could be responsible for the antibiofilm, antivirulence and antibacterial activities towards human pathogenic *S. aureus* as evinced in our study. In a study performed by Divya et al. (2018) in the coral *Favites abdita*, they have documented the ability of biofilm forming opportunistic pathogen *Staphylococcus sciuri* on multiple coral hosts.

As future prospects, the study can be further extended to identify the specific targets involved in the QS system of *S. aureus* which could be inhibited/interfered by the active molecules produced from the coral-associated *P. aeruginosa* CBMGL12 through cloning and expression of *S. aureus* associated QS components in genetic model systems and careful observation of anti-QS activity. Also, the production of these antivirulence metabolites by *P. aeruginosa* CBMGL12 shall be over enhanced using the cheapest carbon and nitrogen sources to commercialize these bioactive molecules cost-effectively. The pathways in which *P. aeruginosa* CBMGL12 synthesized these bioactive molecules can also be a choice of study for the future young minds.

## Data Availability Statement

The datasets presented in this study can be found in online repositories. The names of the repository/repositories and accession number(s) can be found in the article/Supplementary Material.

## Author Contributions

JS and GK conceived the study, designed, supervised the research, and prompted the manuscript. KV performed *in vitro* experiments, analyzed the data, and prepared the manuscript. SD and RD assisted sample collection and *in vitro* studies. KT and ST assisted data collection and analysis. All authors have read and approved the manuscript.

## Conflict of Interest

The authors declare that the research was conducted in the absence of any commercial or financial relationships that could be construed as a potential conflict of interest.

## Abbreviations

BIC: biofilm inhibitory concentration
CA-MRSA: community acquired-methicillin resistant *Staphylococcus aureus*
CBMGL: coral-associated bacteria from microbial genomics laboratory
CLSI: Clinical Laboratory Standards Institute
EtOAc: ethyl acetate
FAME: fatty acid methyl esters
FT-IR: fourier transform infrared spectroscopy
FT-NMR: fourier transform-nuclear magnetic resonance
GC-MS: gas chromatography-mass spectrometry
HPLC-UV: high performance/pressure liquid chromatography-UV detection
MBC: minimum bactericidal concentration
MDR: multidrug resistant
MHA: mueller-hinton agar
MHB: mueller-hinton broth
MIC: minimum inhibitory concentration
MS/MS: tandem mass spectra
MTCC: microbial type culture collection, India
MTP: microtitre plate
NIST: The National Institute of Standards and Technology
QQ: quorum quenching
QS: quorum sensing
RBCs: red blood cells
UV-Vis: Ultraviolet-visible spectrophotometry.
