# Supplementary material for: Fatty Acid Methyl Esters From the Coral-Associated Bacterium Pseudomonas aeruginosa Inhibit Virulence and Biofilm Phenotypes in Multidrug Resistant Staphylococcus aureus: An in vitro Approach
Source: Front Microbiol. 2021 Mar 23;12:631853. doi: 10.3389/fmicb.2021.631853 (PMC8023275; doi:10.3389/fmicb.2021.631853)
Supplement: Supplementary file 1 [file Data_Sheet_1.pdf]

SUPPLEMENTARY DATA

Fatty acid methyl esters from the coral-associated bacterium *Pseudomonas aeruginosa* inhibits virulence and biofilm phenotypes in multidrug resistant *Staphylococcus aureus*: An in vitro approach

Vijay Karuppiah[1,3], G Seghal Kiran[2], S Divya[1], Kavitha Thangavel[3], Sathiamoorthi Thangavelu[3], Ranjithkumar Dhandapani[3], Joseph Selvin[1]*

[1]Department of Microbiology, School of Life Sciences, Pondicherry University, Puducherry, India.

[2]Department of Food Science and Technology, School of Life Sciences, Pondicherry University, Puducherry, India.

[3]Department of Microbiology, School of Biological Sciences, Alagappa University, Karaikudi, Tamil Nadu, India.

Correspondence Author
Prof. Dr. Joseph Selvin Ph.D., FNAAS,
Head,
Department of Microbiology,
School of Life Sciences,
Pondicherry University (A Central University),
Puducherry, India.
Email: josephselvinss@gmail.com

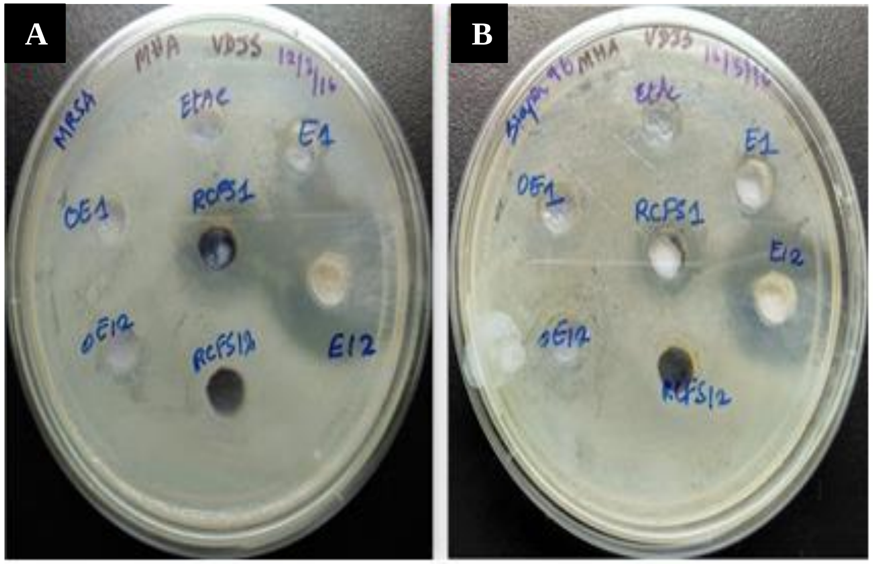

Suppl. Figure 1 Antibacterial activity of the extracts against *S. aureus* strains. MHA plates show the antibacterial activity of ethyl acetate extracts CBE12 against CA-MRSA (A) and MTCC96 (B). Control wells were loaded with ethyl acetate.

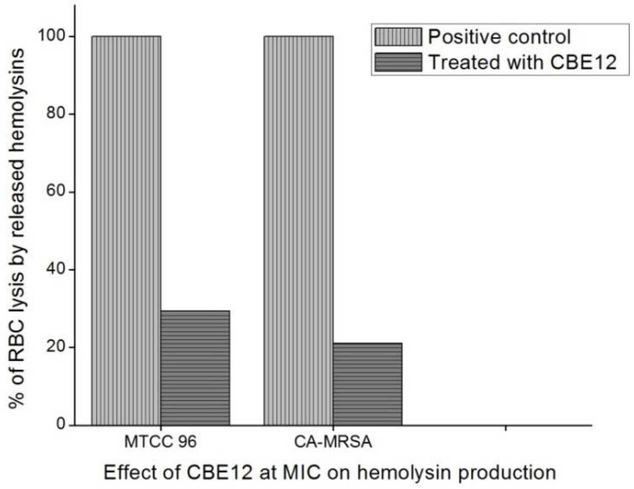

Suppl. Figure 2 Total Hemolysis Assay. Percentage of RBC lysis as estimated by released Hemoglobin OD at 530 nm in control and CBE12 treated *S. aureus* strains.

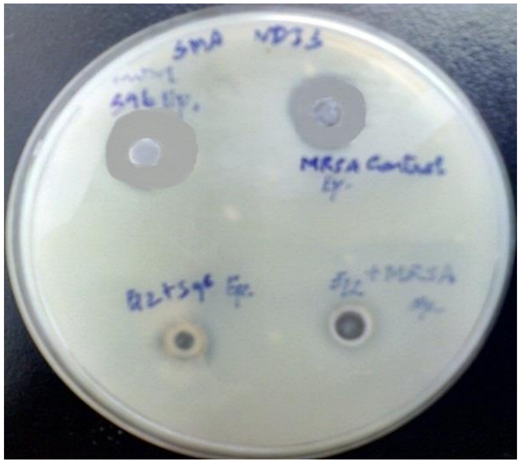

Suppl. Figure 3 Skim milk agar wells loaded with extracellular products (ECP) from *S. aureus* MTCC96 and CA-MRSA. Inhibition of protease production indicated by the decreased zone of casein hydrolysis compared to the controls (without CBE12 treatment).

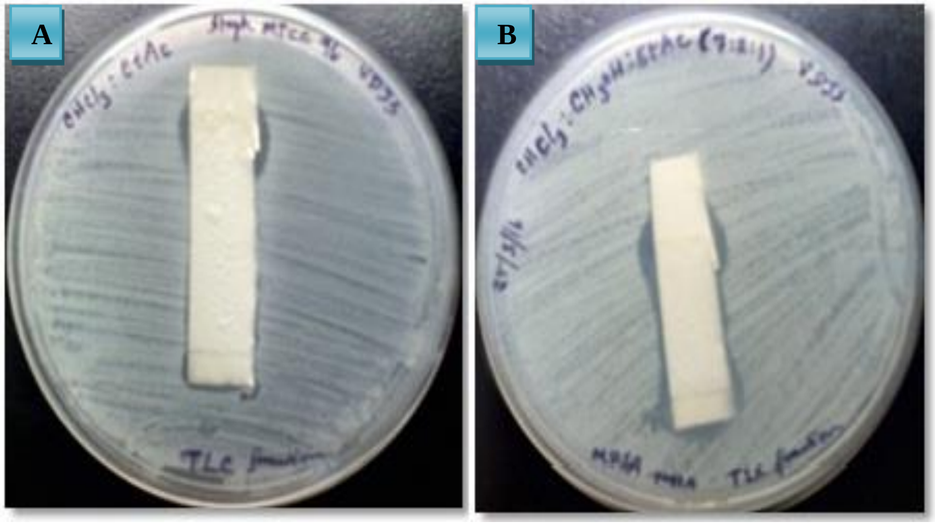

Suppl. Figure 4 Antibacterial activity of compounds in the coral associated bacterial extract CBE12 separated on TLC plates against MTCC96 (A) and CA-MRSA (B).

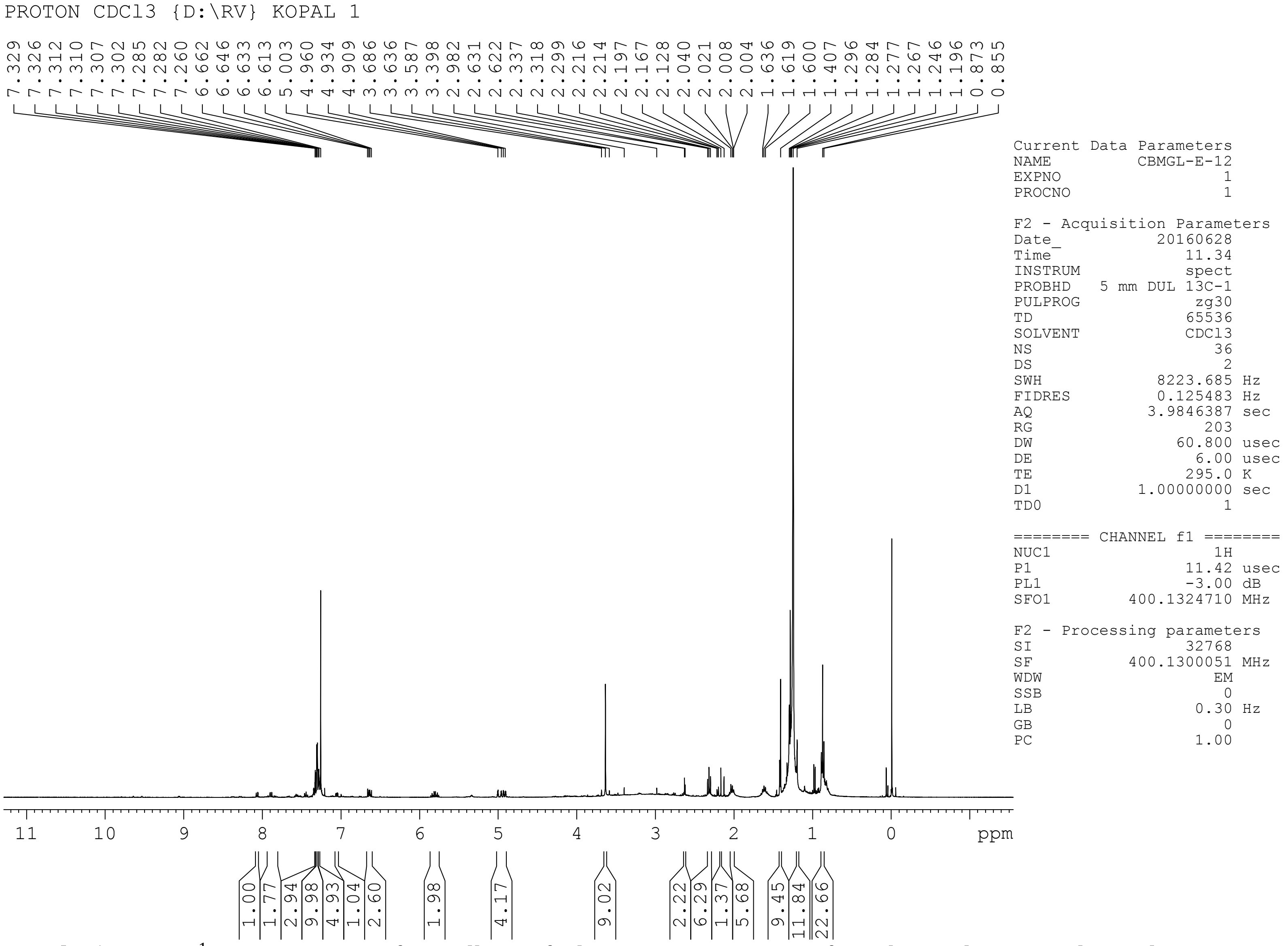

Suppl. Figure 5 H^1 NMR spectrum of partially purified active extract CBE 12 from the coral associated *Pseudomonas aeruginosa* strain CBMGL12

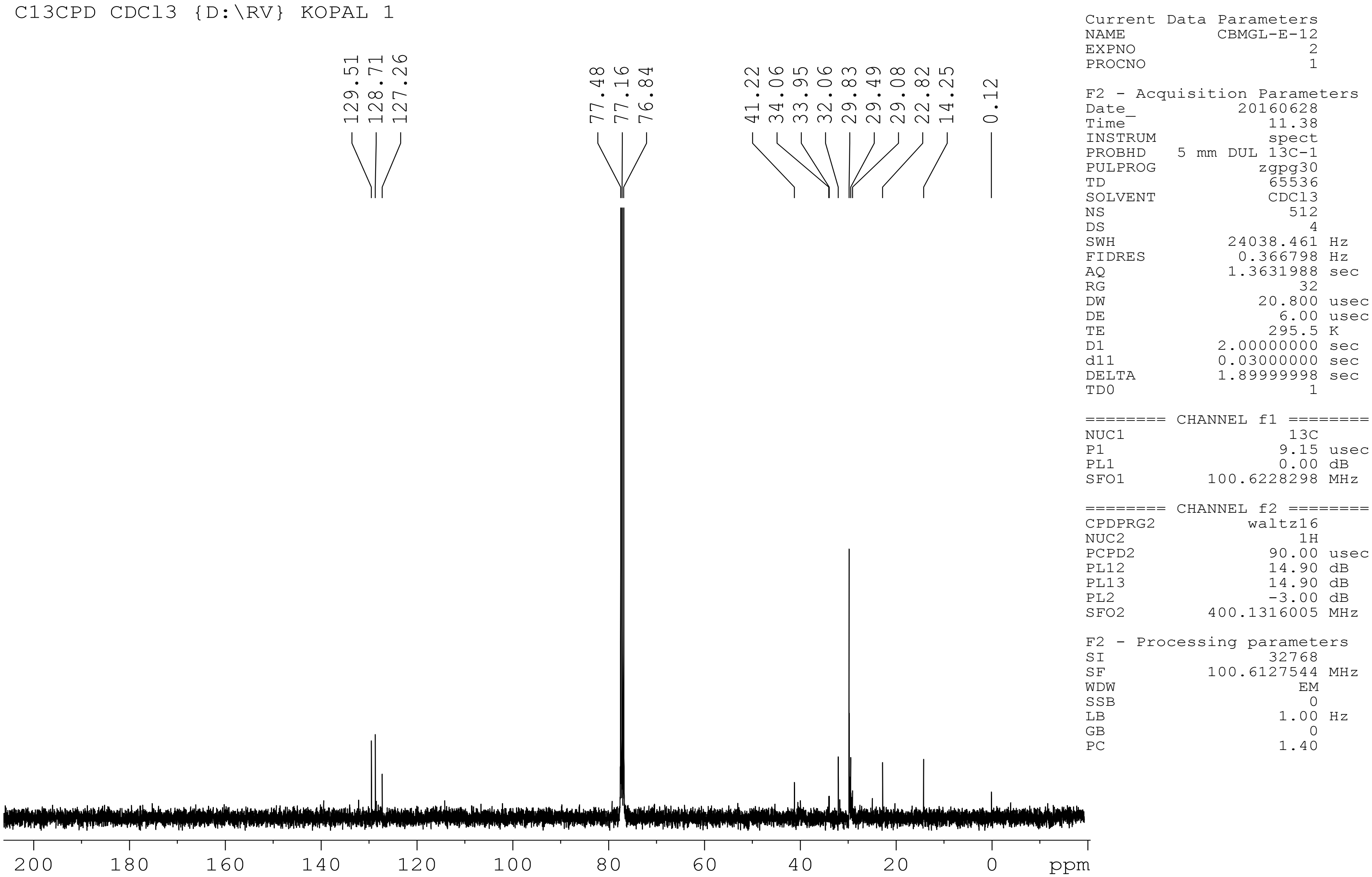

Suppl. Figure 6 C^{13} NMR spectrum of partially purified active extract CBE 12 from the coral associated *Pseudomonas aeruginosa* strain CBMGL12

Suppl. Table 1 Biochemical identification of the producer bacterium *Pseudomonas aeruginosa* strain CBMGL12

Characteristics	Observation
Gram Staining	Gram Negative Rods
Catalase	Positive
Oxidase	Positive
Nutrient agar	Green colonies
Cetrimide agar	Luxuriant yellowish Green with fluorescence
TCBS agar	Bluish green colonies
EMB agar	No growth
Motility	Motile

Suppl. Table 2 Percentage of hemolysin inhibition in MTCC 96 by HPLC fractions

Fraction ID	Hemolysin inhibition (%)
FA1	5.52
FA2	0.0
FB1	6.5
FB2	0.0
FC1	6.72
FC2	0.0
FD1	7.22
Column fraction(CFA2)	5.77

Suppl. Table 3 Percentage of hemolysin inhibition in CA-MRSA by HPLC fractions

**Fraction ID**	**Hemolysin inhibition (%)**
FA1	16.42
FA2	11.58
FB1	16.23
FB2	11.98
FC1	11.95
FC2	12.42
FD1	11.39
Column fraction (CFA2)	14.48
